# A Novel Semi-quantitative In Vitro Evaluation of Gelling Fiber Dressings: The Microcontouring Method

**DOI:** 10.7759/cureus.101065

**Published:** 2026-01-08

**Authors:** Sophie Ballamy, Donna Kesteven, Samantha Hutchinson, Lauren Bagshaw

**Affiliations:** 1 Advanced Wound Care, Research and Development, Convatec Ltd., Deeside, GBR; 2 Research and Development, Convatec Ltd., Deeside, GBR

**Keywords:** conformability, gelling fiber dressing, imagej, imaging, intimate contact, microcontouring, sodium carboxymethylcellulose, test method, wound care

## Abstract

The ability of a dressing to adapt to the wound surface upon hydration is critical for maintaining a moist wound-healing environment and for minimizing any "dead space" between the dressing and the wound surface, where pathogens may proliferate. This ability to conform to a surface can be affected by many factors, including (but not limited to) dressing composition, fiber directionality, and any additional features, such as strengthening stitches, additional layers, or welding. Gelling fiber dressings, such as those composed of sodium carboxymethylcellulose, form a gel upon hydration, promoting a moist healing environment and sequestering harmful wound components. However, there is currently no standardized method to assess a dressing’s ability to conform to contoured wound surfaces.

This technical report describes a novel in vitro method (referred to as the microcontouring method) to semi-quantitatively evaluate dressing conformity. A 3D-printed test rig with a contoured sample platform and integrated fluid port was developed. Samples of one type of carboxymethyl-cellulose-based gelling fiber dressing were hydrated with isotonic simulated wound fluid under controlled conditions (n=13). Time-lapse video and endpoint still images were captured to assess dressing behavior and quantify residual "dead space" (measured area of gaps between sample and rig surface) using ImageJ software (Bethesda, MD: National Institutes of Health). The method demonstrated strong reproducibility, with a coefficient of variation below 10% across three analysts. The setup enabled clear visualization of hydration dynamics and allowed for semi-quantitative comparison between samples. The microcontouring method provides a reproducible, semi-quantitative approach for evaluating the conformability of gelling fiber dressings. While further work is required to determine reproducibility across a wider range of gelling fiber dressings, this proof-of-principle work offers a potentially valuable tool for product development and comparative testing, addressing a current gap in standardized assessment techniques.

## Introduction

Gelling fiber dressings, such as those composed of sodium carboxymethylcellulose, are widely used in wound care due to their ability to absorb wound exudate and form a cohesive aqueous gel [1]. This gelled dressing promotes a moist wound environment - a well-established factor in optimal wound healing [2,3]. As the dressing hydrates, it sequesters wound fluid within its fiber matrix [4], effectively trapping potentially harmful components, such as proteolytic enzymes, matrix metalloproteinases, and bacteria. Poor management of such factors may lead to unfavorable clinical outcomes, such as increased infection risk, delayed healing, and/or peri-wound maceration [5].

A critical feature of gelling fiber dressing performance is its ability to achieve intimate contact with the wound bed - a phenomenon referred to as microcontouring. This close conformity minimizes the formation of voids or "dead space" that can contribute to tissue maceration and the proliferation of pathogens [6,7].

Historically, microcontouring assessments for gelling fiber dressings have relied on meat-based substrates to simulate wound surfaces [6,8]. While visually illustrative, these substrates suffer from low reproducibility due to the inherent variability of biological tissues, rendering them unsuitable for quantitative analysis. To address this limitation, a custom-designed, 3D-printed solid surface with an integrated fluid port was developed. This standardized platform enables more consistent and reproducible evaluation of dressing performance under controlled in vitro conditions.

This technical report describes a novel in vitro method (referred to as the microcontouring method) for assessing the ability of gelling fiber dressings to conform to a contoured surface during hydration. The test setup uses the 3D-printed platform and isotonic simulated wound fluid (solution A: sodium/calcium chloride, tinted with blue food dye) to challenge the dressing against a surface of a fixed depth and shape [9].

The method produces the following two outputs: (1) time-lapse video footage capturing the full hydration process, providing visual insight into dressing behavior, and (2) a still image at the endpoint, from which a semi-quantitative measurement of any remaining dead space can be derived. Time-lapse footage serves as both a visual aid and contextual evidence for the behavior of the dressing as it hydrates. The semi-quantitative measurement enables more objective comparisons between dressings of varying compositions, reducing reliance on subjective visual assessment alone. This report outlines the microcontouring method in detail, including the experimental setup details, image analysis, and assessment of its statistical robustness. The primary objective was to validate the setup and workflow.

## Technical report

Equipment

**Figure 1:**
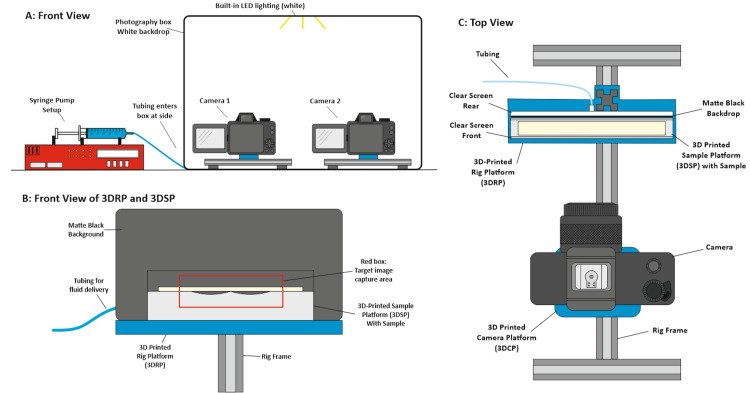
Rig assembly diagrams (multiple views). (A) Front benchtop view showing whole rig setup, (B) zoomed in front view of 3DRP, 3DSP, and matte black backdrop, (C) top view of frame structure comprising 3DCP, 3DRP, and 3DSP. This diagram was created by the authors of this study using CorelDRAW 2019 (Ottawa, Canada: Corel Corp.). 3DCP: 3D-printed camera platform; 3DRP: 3D-printed rig platform; 3DSP: 3D-printed sample platform

To standardize the test setup, a custom-built rig was developed, integrating a 3D-printed rig platform (3DRP) and 3D-printed camera platform (3DCP) into a single adjustable frame (Figures 1A-1C). This configuration enabled precise alignment and fixation in accordance with test requirements. The 3D-printed sample platform (3DSP) was designed to accommodate a 15×90 mm dressing sample and features a customizable contoured surface with a central fluid port. The rig setup may be optionally duplicated to increase testing throughput.

As shown in Figure 1B, the red box highlights the target image capture area, which served as a reference for setting both the camera distance and 3DRP height. The 3DRP was positioned such that the camera view was level with the base of the 3DSP wells, ensuring an unobstructed view through the rig to the black backdrop. Clear acrylic screens are affixed to each side of the 3DSR, serving as windows that allow visualization through the rig and prevent fluid from escaping the system as the sample hydrates. Once the camera and 3DSP positions were optimized, they were held constant for the duration of testing to ensure data comparability.

Sample hydration

**Figure 2:**
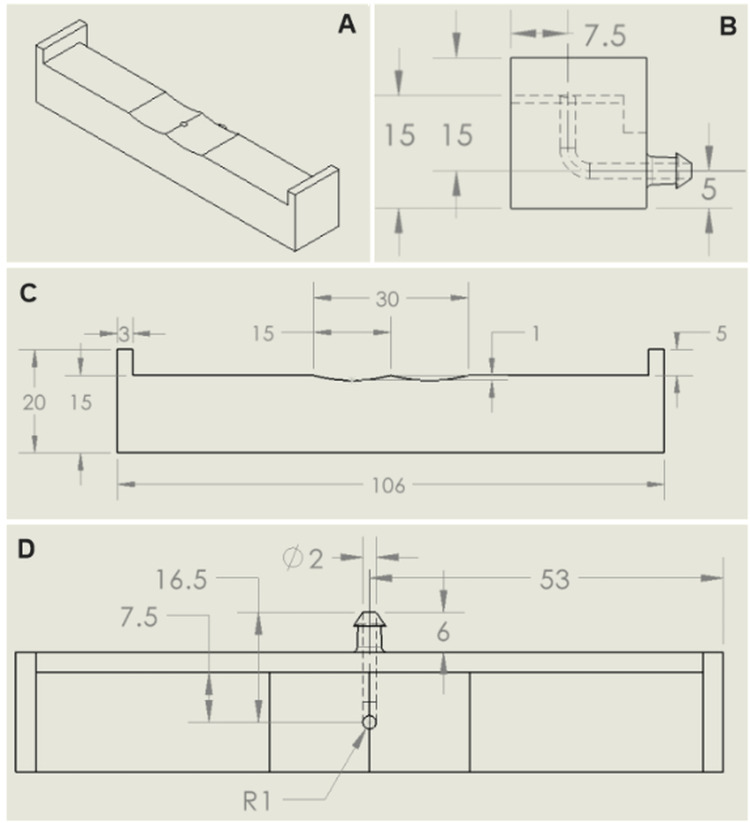
SolidWorks drawings of an example 3DSP (without acrylic screens). The image shows (A) isometric view, (B) right view showing internal fluid pathway, (C) front view, and (D) top view showing port and internal fluid pathway. The figure was generated from a SolidWorks (Dassault Systèmes: Vélizy, France) drawing of an example 3D-printed sample platform; all units are in millimeters. The 3D model and subsequent drawing were created by and are attributed to the author. 3DSP: 3D-printed sample platform

The 3DSP had an integrated fluid port and fluid channel, enabling hydration of each sample from beneath a central point (Figures 2A-2D). This setup allowed for real-time observation of dressing behavior during hydration.

A syringe pump was used to deliver fluid at a constant rate over a fixed time period. During method development, a nominal flow rate of 2.2 mL/h for 1 h was determined to be optimal, as it allowed the gelling fiber samples to become sufficiently hydrated without flooding. The 1-h duration also supported high-throughput testing while yielding measurable results. These parameters may be adjusted as needed, provided all replicates are tested under identical conditions. For future development, it may be beneficial to select a clinically justified flow rate based on current literature and then adjust the test duration accordingly to ensure the sample is sufficiently saturated.

Image capture

To account for potential fiber directionality, each sample was assessed in two orthogonal directions (90° apart), aligned with the dressing’s edges. Data from both orientations were pooled to provide a representative overall assessment of each product. Prior to testing, the fluid tubing was primed, and the 3DSP was dried. The sample was then placed centrally and flat on the surface, ensuring it remained horizontal and undeformed.

**Figure 3:**
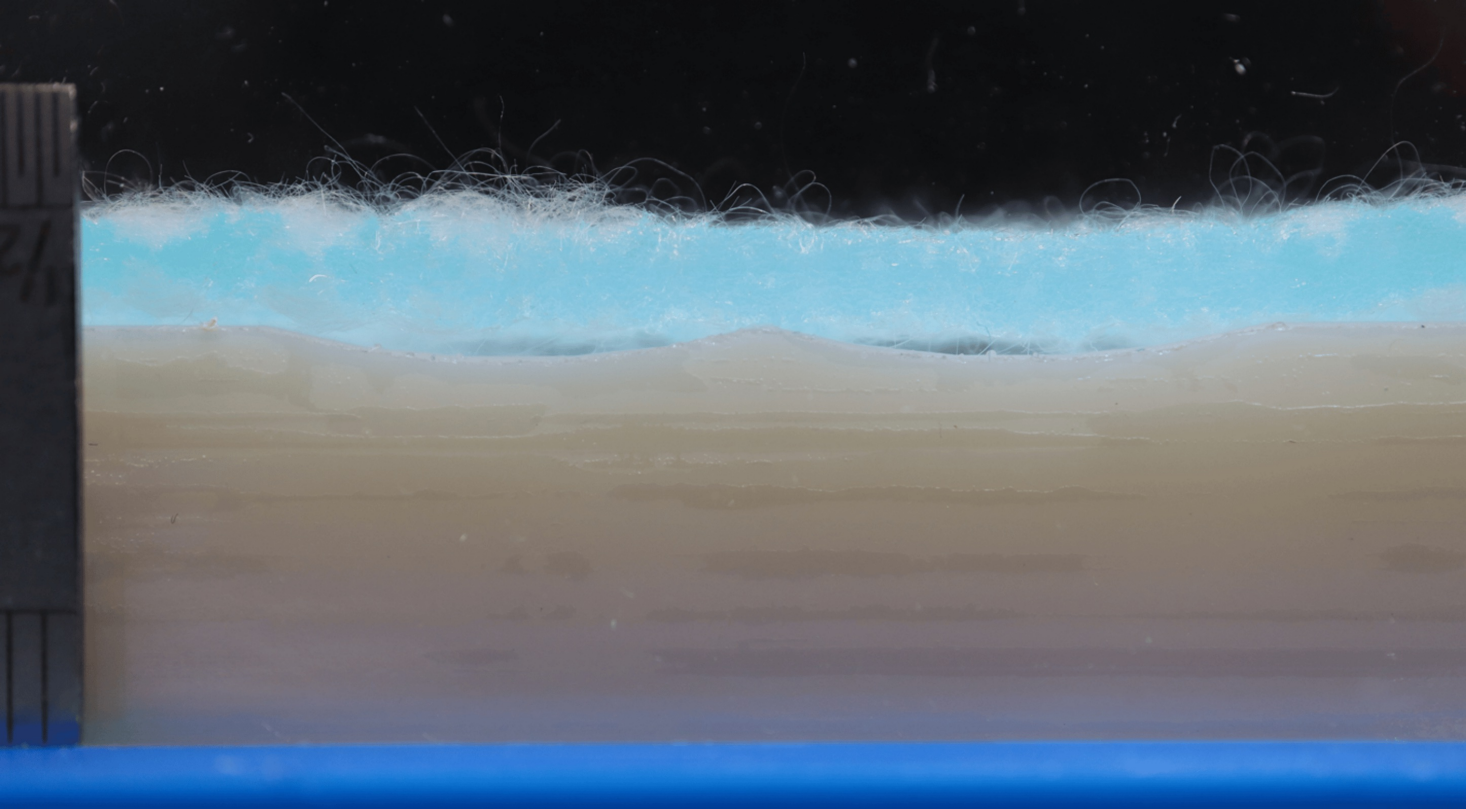
Example of test endpoint. Image taken with calibrated rule in frame for scale.

The camera, set to time-lapse mode, was focused to ensure a clear and sharp image (Table 1). The camera, syringe pump, and timer were started simultaneously (as close together as practicable) and run for the predetermined duration. At the endpoint, samples should appear saturated within the camera frame; full saturation across the entire sample is not necessary. A still image was captured at the endpoint with a calibrated ruler in frame to provide scale for analysis (Figure 3). An example of time-lapse video output is shown in Video 1.

**Table 1:** Example camera configuration and parameters. 3DSP: 3D-printed sample platform; HD: high definition; LED: light-emitting diode

Item/factor	Description
Camera	Canon EOS Mark II (Tokyo, Japan: Canon Inc.)
Lens	Canon EF-M 28 mm macro lens, set to standard macro setting
Time-lapse settings	Full-HD timelapse mode, 1 shot every 5 sec. Auto-exposure: fixed first frame
Image capture	Full auto mode with calibrated scale in frame
Housing	PiXAPRO 70 cm foldable LED light tent (PiXAPRO: West Midlands, UK) with white backdrop installed
Contrast/backdrop	Matte black card used directly behind 3DSP

**Video 1:** Time-lapse footage example.

Image analysis

Semi-quantitative analysis was performed using image analysis software ImageJ (ImageJ.net) (Bethesda, MD: National Institutes of Health) on the endpoint image for each replicate. The measurement area for this analysis used the front view of the 3DSP, as in the final image (Figure 3). Hydrated samples conformed to the contoured surface to varying degrees, and any non-conforming (i.e., uncontacted) area was measured. A high-contrast backdrop (e.g., matte black) was used to clearly differentiate the sample and the 3DSP from the measurement area (Figures 1A-1C, 2A-2D, 3). The initial open area (between the dry, horizontal sample and the contoured surface) was estimated based on the 3DSP’s 3D model. The difference between the starting area and the endpoint area was calculated and expressed as a percentage of the starting area - this can be used to indicate the degree to which the sample has conformed to the rig, i.e., 100% means the sample has fully conformed to the rig surface, 0% means the sample has not conformed at all and has remained unchanged from the starting state.

\( \textbf{Percentage area conformed (%) =}\left\lfloor \frac{\textbf{Starting area - endpoint area}}{\textbf{Starting area}} \right\rfloor × 100 \)

**Figure 4:**
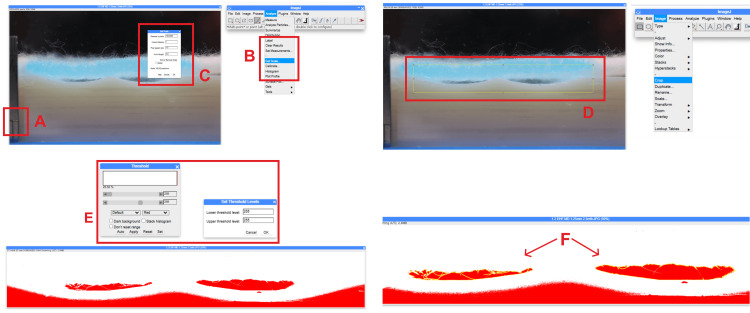
Image analysis process using ImageJ. The image shows (A) line drawn from which to set scale, (B) "set scale" option in analysis menu, (C) set scale dialog box, (D) crop area, (E) threshold dialog box, and (F) "dead space" areas encircled in yellow are included for measurement. ImageJ (Bethesda, MD: National Institutes of Health)

In ImageJ, the image was opened, the ruler was used to set the scale (Figures 4A-4C), the image was cropped to the measurement area (Figure 4D), converted to 8-bit, and thresholded to isolate the measurement region (Figure 4E). The "magic wand" tool was used to select the target measurement area, and the area was measured using the "analyze" function (Figure 4F).

Method robustness and reproducibility

To assess the robustness of the image analysis method, a single image taken from method development was analyzed by three independent analysts. Each analyst performed 13 repeated measurements, yielding a total of 39 data points. The coefficient of variation (CV) was used to evaluate data variability. CV is defined as the ratio of the standard deviation to the mean and is expressed as a percentage. In many resources (literature, academia, online), the typical range for CV is calculated as follows.

\( \text{<10% = Excellent (very little variation)}\\\\ \text{10% - 30% = Moderate (generally acceptable variation)}\\\\ \text{>30% = Not acceptable (high variation and potential issues)}\\\\ \)

As shown in Table 2, the CV for each analyst and the pooled data set was below 10%, indicating excellent reproducibility. This indicates that the data showed very little variation. It can therefore be concluded that this microcontouring method and image analysis process are both reproducible and transferable between analysts. For future testing, it would be recommended that each replicate be analyzed by multiple analysts, with repeated measurements per analyst. Pooled data can then be averaged, and the CV calculated to monitor variation. If the CV exceeds 30%, potential sources of error (e.g., analyst error or equipment issues) should be investigated.

**Table 2:** Robustness assessment data overview. Single-image measurement using a 1.00-mm depth rig: area (mm²), with per-analyst and pooled-analyst analysis. CV: coefficient of variation; SD: standard deviation

Measurement number	Analyst 1 (mm²)	Analyst 2 (mm²)	Analyst 3 (mm²)
1	2.011	1.994	2.108
2	2.152	2.172	1.994
3	2.003	2.171	2.051
4	2.026	2.086	2.165
5	2.002	1.979	1.88
6	1.999	2.204	1.739
7	2.011	2.088	1.948
8	2.003	2.174	1.74
9	2.001	2.116	1.609
10	2.003	2.180	1.796
11	1.875	2.114	2.104
12	2.003	2.086	1.838
13	2.003	2.116	1.986
Mean	2.01	2.11	1.92
SD	0.06	0.07	0.17
CV	3%	3%	9%
Mean (pooled)	2.01		
SD (pooled)	0.13		
CV (pooled)	7%		

## Discussion

The aim of this in vitro test report was to establish and detail a novel method for assessing the ability of a gelling fiber dressing to conform to a contoured surface as it hydrates with isotonic simulated wound fluid. Maintaining moisture balance within a wound is critical to healing. As summarized by Bishop et al., excess exudate can degrade tissue and exacerbate wound deterioration, while insufficient moisture may impair healing [2]. The physical properties of primary wound dressing materials are therefore of utmost importance, in that they must absorb and retain excess fluid while preserving a moist interface with the wound bed. Poor management of these characteristics may lead to unfavorable clinical outcomes, such as increased infection risk, delayed healing, and/or maceration of peri-wound skin.

A 2020 consensus document on the management of "the gap" or "dead space" between a dressing and the wound surface stated that treatment should "focus on moisture balance and exudate management, which are critical for effective wound healing" and recommended dressings that "conform to the wound bed" [10]. Despite the clinical importance of this characteristic, existing assessment methods are either qualitative or experimental and lack standardization [8,6]. While previous studies have evaluated wound bed conformity in foam dressings, to our knowledge, the microcontouring method described here represents the first reproducible, semi-quantitative approach for assessing dressing conformity in vitro, specifically for gelling fiber dressings [11,12].

Although this is not a precision assay, the image analysis demonstrated robustness and transferability across analysts, with a low coefficient of variation for the gelling fiber assessed. Future studies will apply this method to a broader range of dressings to fully explore its discriminatory potential. Additional refinements could include automation of image analysis to enhance precision and reduce manual variability; however, this would require the use of specialist software.

As this method requires the dressing sample to be cut to expose a cross-sectional view, the structural integrity of samples not indicated to be cut would need to be considered before testing. For example, products such as composite foam dressings containing unconstrained superabsorbent materials (i.e., loose powders or polymers) are likely to swell significantly and exceed the sample boundary, which would not be representative of routine use, as the sample would not be cut. Such limitations should be considered when selecting dressings for assessment. Overall, the microcontouring method provides a practical and reproducible framework for evaluating dressing conformity, with potential applications in product development, comparative testing, and performance benchmarking.

## Conclusions

Providing a visual representation of a gelling fiber dressing’s ability to hydrate and conform to a contoured surface offers valuable insight into this key physical characteristic and helps illustrate how the dressing may interact with the wound bed. Other methods have relied on subjective interpretation; this novel standardized microcontouring method allows for a robust and transferable approach. By combining time-lapse video for contextual visualization with semi-quantitative measurement of percentage area conformed, this method enables more meaningful and objective comparisons between test samples, provided that the test parameters are identical for all replicates.
